# Prospective randomized controlled trial in the treatment of lateral epicondylitis with a new dynamic wrist orthosis

**DOI:** 10.1186/s40001-018-0342-9

**Published:** 2018-09-15

**Authors:** J. Nowotny, B. El-Zayat, J. Goronzy, A. Biewener, F. Bausenhart, S. Greiner, P. Kasten

**Affiliations:** 1 Orthopaedic-Traumatology Centre (OUC), Carl-Gustav Carus University Dresden, Fetscherstraße 74, 01307 Deutschland, Germany; 2 Department of Orthopaedic Surgery, University Hospital, Marburg, Germany; 3 Department of Orthopaedic Surgery, University Hospital, Tübingen, Germany; 4 Sporthopaedicum, Regensburg, Germany; 5 Orthopaedic-Surgery Centre (OCC), Tübingen, Germany

## Abstract

**Background:**

In the treatment of lateral epicondylitis (LE), the role of a new dynamic wrist orthosis is unclear.

**Patients and methods:**

Patients suffering from a LE longer than 3 months were multicentrically and prospectively randomized into a physiotherapeutic group (PT group) and in a physiotherapy group plus wrist orthosis (PT + O group). Physiotherapy consisted of daily eccentric strengthening exercises under initial professional supervision. Inclusion criteria were a Placzek score greater than 4. Exclusion criteria were previous surgery, rheumatic arthritis, elbow instability, radicular symptoms, higher-grade extensor tendon rupture, or cervical osteoarthritis. The clinical evaluation was performed after 12 weeks and 12 months. The Patient-Rated Tennis Elbow Evaluation (PRTEE) scale, Placzek Score, the pain rating (VAS), range of motion and the Subjective Elbow Score were evaluated.

**Results:**

Of the initially 61 patients, 31 were followed up after 12 weeks and 22 after 12 months. Twenty-nine patients (43%) were male, the mean age was 46, and 44 patients (66%) had the right elbow involved. At 12 weeks, there was a pain reduction on the VAS in both groups (PT + O: 6.5–3.7 [*p* = .001]; PT: 4.7–4.1 [*p* = .468]), albeit it was only significant for the PT + O group. At 12 months, reduction was significant in both groups (PT + O: 1.1 [*p* = .000]; PT: 1.3 [*p* = .000]). The painless maximum hand strength in kg improved in both groups significant after 3 and 12 months. The Placzek score was reduced from 8.25 to 3.5 [*p* = .001] after 12 weeks for the PT + O group and from 8.1 to 3.8 [*p* = .000] in the PT group, as well as after 12 months in the PT + O group to 0 [*p* = .000] and in the PT group to 2.0 [*p* = .000]. The PRTEE improved in both groups after 12 weeks (PT + O: 52.8––31.3 [*p* = .002]; PT: 48.6–37.6 [*p* = .185]) and 12 months (PT + O: 16.15 [*p* = .000]; PT: 16.6 [*p* = .000]), although the reduction at 12 weeks was not significant for the PT group.

**Conclusion:**

The elbow orthosis appears to accelerate the healing process with respect to the PRTEE and pain on the VAS (12 weeks follow-up), although there is an adjustment after 12 months in both groups and a significant improvement of symptoms is achieved in all endpoints.

## Background

The lateral epicondylitis (LE) is a very common disease and is defined as a painful inflammation and/or degeneration of the extensor carpi radialis brevis (ECRB) and the extensor communis digitorum (ECD) at the lateral epicondyle. The first description of this disease was by Runge in 1873. Pathogenetically, the LE usually occurs due to a cumulative mechanical overload which results in metabolic changes and causes microtears. Multiple tears then cause secondary inflammatory and degenerative reactions and in further healing process, a fibroblastic tendinosis develops [1].

The incidence is quantified by 3–5% of the total population with the prevalence in patients between 40 and 59 years with balanced gender relationship [2–4]. The disease is often observed in tennis players (10–15%), in which amateur players are much more affected than professional tennis players, and is therefore, more commonly known as tennis elbow.

There are various classifications of the disease, although the Nirschl et al. classification is the most common [5]. Table 1 gives an overview of the four stages.

**Table 1:** Classification of lateral epicondylitis according to Nirschl et al. [11]

Stage	Characteristics
I	Acute inflammation, which can spontaneously heal without residues
II	Tendinosis of the tendon with angiofibroblastic hyperplasia, vascular hyperplasia and unorganized collagen
III	Partial or complete rupture of the tendons
IV	Fibrosis, soft tissue calcifications and osseous calcifications

Clinically, there is usually a significant load-dependent pain at the lateral epicondyle with pain in the upper and lower arm combined with weakness in the wrist. The beginning of symptoms is mostly slow, but can also occur abruptly. The diagnosis of LE is mainly based on clinical examination, with localized pressure on the radial epicondyle typically causes pain. In addition, isometric extension or passive flexion of the wrist or of the middle finger with extended elbow causes pain (Thompson test, Mill’s test, and Maudsley’s test). The “Patient-Rated Tennis Elbow Evaluation” (PRTEE) can classify clinical symptoms, which involves questions about pain and malfunction in daily activities [6, 7]. The range of motion is usually not affected. In case of atypical or refractory pain potential, differential diagnoses such as a hypertrophic and painful plica humeroradialis, chondropathy of the elbow joint, osteoarthritis, loose body of the joint or an osteochondritis dissecans, extended diagnostics can be performed with X-ray and, e.g., magnetic resonance imaging (MRI). In addition, ultrasound can be a useful tool for diagnostic of LE. Besides structural changes affecting the tendon, also neo-vascularization can be detected by color Doppler exploration. However, MRI is more reproducible, can show damages or detachment of the ECRB tendon from the lateral epicondyle and gives more information about potential intra-articular pathology [8].

The treatment of the LE is manifold. It ranges from stress reduction on the epicondyle, immobilization of the elbow, physiotherapy (eccentric exercises, transverse friction), oral non-steroidal anti-inflammatory drugs, locally applied drugs (cortisone, platelet-rich plasma [PrP], hyaluronic acid) and different surgical techniques (open or arthroscopic) to supply with orthoses [9–15]. There are several types of bandages and orthoses that are used in the treatment of LE, which should frequently provide a “counterforce” to reduce the load on the extensor tendons [16, 17]. First, there are the classic bandages/elbow sleeves, which are usually used as a stabilization of the elbow. Elbow braces/clasps have a pad which is placed distal to the lateral epicondyle to compress very locally at the insertion, and therefore, reduces the forces on the common extensor tendon. Furthermore, there are wrist orthoses which should reduce the overload on the wrist extensors. However, the reported effects of those orthoses in LE are heterogeneous and there are limited studies that compare different orthotic devices against each other or a placebo with a high evidence level [18–20]. There are no studies that evaluate the effect of a dynamic wrist orthoses. The aim of the current study is, therefore, the prospective randomized evaluation of a new dynamic wrist orthoses in the treatment of LE as compared to physiotherapy alone.

## Methods

For the reporting of this study, the Consolidated Standards of Reporting Trials (CONSORT) 2010 statement, the Template for Interventions (TIDieR) and the Consensus on Exercise Reporting Template (CERT) were used [21, 22]. The study was performed in accordance with the ethical standards World Medical Association Declaration of Helsinki (2002).

### Study design

The study was a multicentre (three specialized shoulder and elbow centres in Germany), prospective, randomized controlled trial. The data collection and the randomisation were done via an external database (REDCap—Research Electronic Data Capture). Ethical approval was obtained from the Ethical Committee (EK 16012014) and the ethical standards in science research were respected [23].

### Participants

In three different specialized shoulder/elbow centres, patients were prospectively included in the time period from December 2013 to March 2016. Inclusion criteria were symptoms for more than 3 months, a Placzek score [24] greater than 4 and written consent of the patient. Exclusion criteria were previous surgery, rheumatic arthritis, elbow instability, former fracture of the elbow, higher-grade extensor tendon ruptures (more than 50%) or a cervical radiculopathy. The clinical evaluation was performed before intervention and after 12 weeks and 12 months.

### Intervention

**Fig. 1:**
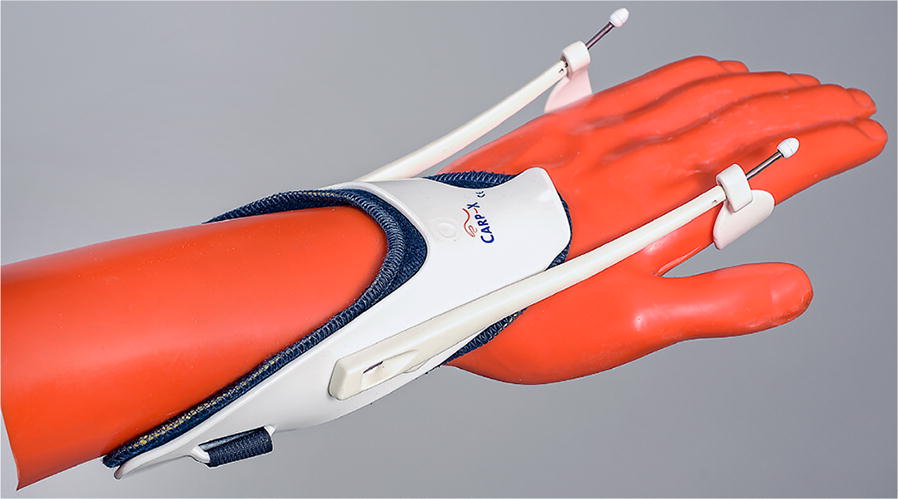
Novel dynamic wrist orthosis that unloads wrist extensors

Consecutively involved patients were randomized into two groups: the first group received physiotherapy (PT group) and the second a dynamic extension wrist orthosis (dynamic epicondylitis orthosis, CARP-X, Sporlastic GmbH, Nürtingen, Germany) (Fig. 1) plus physiotherapy (PT + O group). The physiotherapy consisted of daily eccentric strengthening exercises (three times a day for 10 min). Therefore, the elbow-extended forearm was placed at a flat base, so that the wrist is free in mobility. The opposite, not affected hand was placed on the affected hand and a slight pressure was given backwards. After holding the position for 3 s, the affected hand was transferred to the flexion position for slight eccentric strengthening. This was repeated ten times. For standardization, all patients received the same exercise sheet with written and pictorial description. Initial supervision was provided by a professional physiotherapist in six sessions. The number and duration of exercises was recorded at the 12 weeks follow-up.

### Outcomes

Demographic data such as age, gender, weight, handedness and profession were collected. Pain was determined using the visual analog scale. The grip strength (painless and maximum) was measured with fixed elbow on the table by an electronic hand-held dynamometer (TL-LSC 100, Liteexpress GmbH Coesfeld, Germany), wherein the painless grip strength measurement was performed first. Furthermore, range of motion (ROM) with flexion, extension, pronation and supination was measured with a manual goniometer.

The “Patient-Rated Tennis Elbow Evaluation” (PRTEE) scale [7], Placzek Score [24], the pain rating on visual analog scale (VAS) and the “subjective elbow value” (SEV) [25] were evaluated. All outcome parameters were recorded at baseline, 12 weeks and 12 months.

### Statistical analysis

Statistical analysis was performed with SPSS Statistics software (version 24; IBM, Armonk, NY, USA) for descriptive statistics, with significance level at *p* < .05, while all data were presented as mean with standard deviation. The differences between baseline and postinterventional values were evaluated using the “paired *t* test”.

## Results

**Fig. 2:**
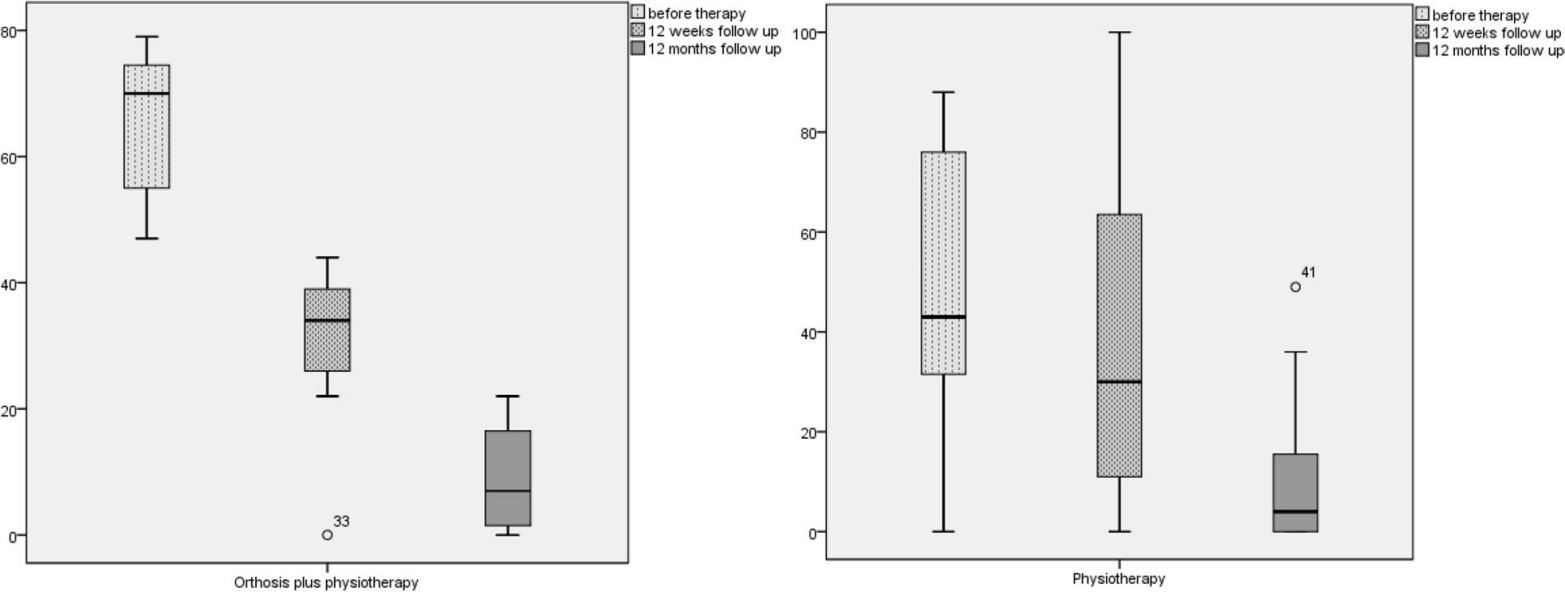
Boxplot of outcome parameter—pain on the VAS: baseline, 12 weeks and 12-month follow-up, left: orthosis plus eccentric strengthening exercises, right: eccentric strengthening exercises alone. (PT + O: 6.5–3.7 [*p* = .001] to 1.1 [*p* = .000]; PT: 4.7–4.1 [*p* = .468] to 1.3 [*p* = .000])

Of the initially involved 61 patients, 31 were followed up after 12 weeks and 22 after 12 months, so there is a dropout rate of about 50% for the 12 weeks and approximately two-thirds during the 12 months follow-up with no difference between the groups. An overview can be found in Table 2. Twenty-nine patients (43%) were male, the mean age was 46, and 44 patients (66%) had the right elbow involved. All outcome parameters were compared between the baseline and the follow-up at 12 weeks and 12 months. There was no difference between the two groups in respect to all baseline data in regard with age [*p* = .102], gender [*p* = .397], handedness [*p* = .693], Placzek score [*p* = .154], PRTEE score [*p* = .519], hand strength (painless and maximum) [*p* = .678 and *p* = .636], pain on VAS [*p* = .312] and range of motion (ROM) (Table 2). There was a significant pain reduction on the VAS after 12 weeks only in the (PT + O group: 6.5–3.7 [*p* = .001]; PT: 4.7–4.1 [*p* = .468]). After 12 months, reduction was significant in both groups (PT + O: 1.1 (SD: 1.0) [*p* = .000]; PT: 1.3 (SD: 1.6) [*p* = .000]) (Fig. 2). The SEV increased in both groups after 12 weeks (PT + O: 36–63 [*p* = .000]; PT: 47–57 [*p* = .190]).

**Table 2:** Summary of results in dependence of therapy group

Score	Physiotherapy group (PT group)			Physiotherapy and orthosis group (PT ± O group)			*p* value baseline
	Baseline (*n* = 33)	12 weeks (*n* = 15)	12 months (*n* = 12)	Baseline (*n* = 28)	12 weeks (*n* = 16)	12 months (*n* = 10)	
Age (years)	46			47			.102
Male	10 (30%)			16 (44%)			.397
Handedness right	29 (88%)			26 (89%)			.693
Pain (VAS)	4.7 (SD: 2.8)	4.1 (SD: 3.1) [*p* = .468]	1.3 (SD: 1.6) [*p* = .000]	6.5 (SD: 1.7)	3.7 (SD: 2.6) [*p* = .001]	1.1 (SD: 1.0) [*p* = .000]	.312
Flexion	127 (SD: 19)	130 (SD: 21) [*p* = .175]	131 (SD: 17) [*p* = .734]	133 (SD: 19)	138 (SD: 11) [*p* = .361]	135 (SD: 9) [*p* = .356]	.399
Extension	7 (SD: 14)	4 (SD: 14) [*p* = .175]	9 (SD: 21) [*p* = .714]	2 (SD: 4)	1 (SD: 2) [*p* = .271]	0 (SD: 0) [*p* = .689]	.704
Maximum hand strength in kg	23.8 (SD: 17.3)	26.7 (SD: 16.7) [*p* = .051]	33.7 (SD: 14.5) [*p* = .061]	20.4 (SD: 16.5)	20.6 (SD: 12.5) [*p* = .943]	26.9 (SD: 9.9) [*p* = . 889]	.636
Painless hand strength in kg	14.8 (SD: 17.5)	19.9 (SD: 17.1) [*p* = .031]	32.2 (SD: 15.9) *p* = .013]	9.9 (SD: 12.1)	18.9 (SD: 14) [*p* = .009]	25.3 (SD: 9.3) [*p* = .028]	.678
Placzek score	8.1 (SD: 1.76)	3.8 (SD: 2.98) [*p* = .000]	2 (SD: 2.5) [*p* = .000]	8.25 (SD: 1.84)	3.5 (SD: 2.75) [*p* = .001]	0 (SD: 0) [*p* = .000]	.154
PRTEE score	48.6 (SD: 19.7)	37.6 (SD: 24.1) [*p* = .185]	16.6 (SD: 16.1) [*p* = .000]	52.8 (SD: 16.0)	31.3 (SD: 8.2) [*p* = .002]	16.15 (SD: 16.1) [*p* = .000]	.519

**Fig. 3:**
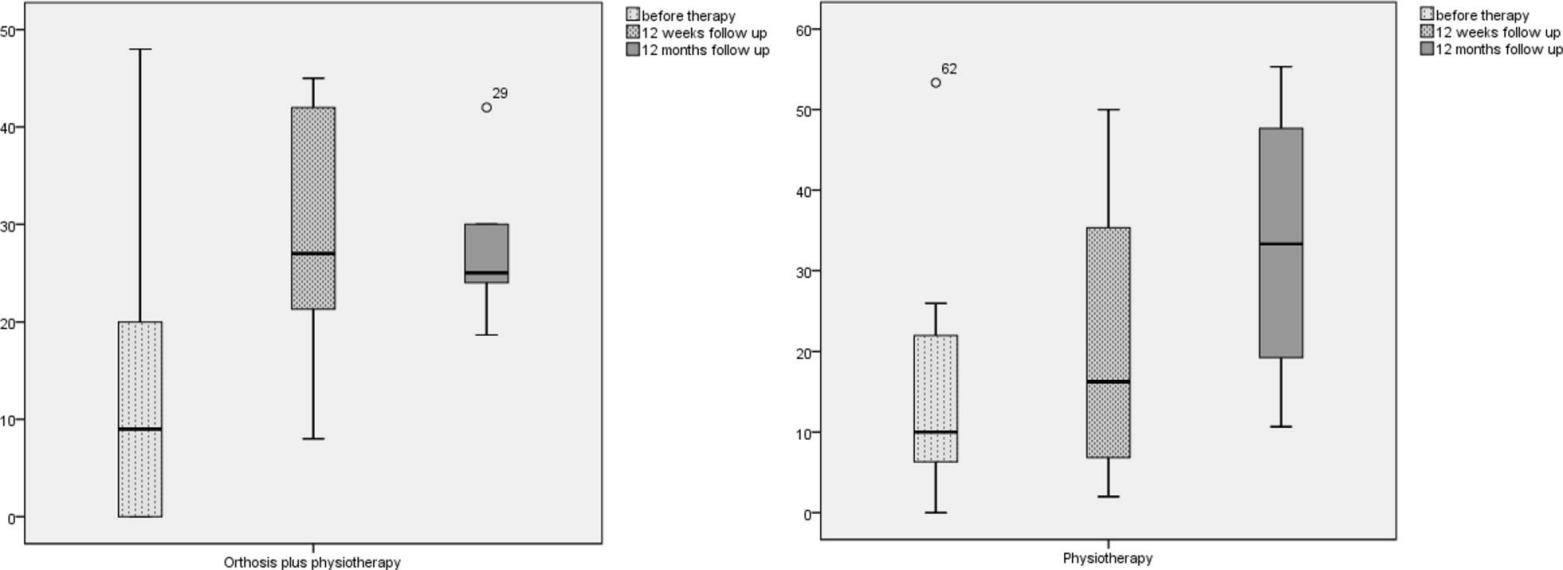
Boxplot of outcome parameter—painless maximum hand strength in kg: baseline, 12 weeks and 12-month follow-up, left: orthosis plus eccentric strengthening exercises, right: eccentric strengthening exercises alone. {PT + O: 9.9 (SD: 12.1) to 18.9 (SD: 14) [*p* = .009] to 25.3 (SD: 9.3) [*p* = .028]; PT: 14.8 (SD: 17.5) to 19.9 (SD: 17.1) [*p* = .031] to 32.2 (SD: 15.9) [*p* = .013]}

The painless maximum hand strength in kg improved in both groups significantly after 12 weeks (PT + O: 9.9 (SD: 12.1) to 18.9 (SD: 14) [*p* = .009]; PT: 14.8 (SD: 17.5) to 19.9 (SD: 17.1) [*p* = .031]) and in the 12 months follow-up (PT + O: 25.3 (SD: 9.3) [*p* = .028]; PT: 32.2 (SD: 15.9) [*p* = .013]) (Fig. 3). The mean values of maximum hand strength in kg increased only slightly in the PT + O group, but there was a stronger trend in the physiotherapy group (PT + O: 20.4 (SD: 16.5) to 20.6 (SD: 12.5) [*p* = .943]; PT: 23.8 (SD: 17.3) to 26.7 (SD: 16.7) [*p* = .051]), although other changes were not significant. At the 12-month follow-up, maximum hand strength in the PT + O group were 26.9 (SD: 9.9) [*p* = .889], as compared to the physiotherapy group with 33.7 (SD: 14.5) [*p* = .061]).

**Fig. 4:**
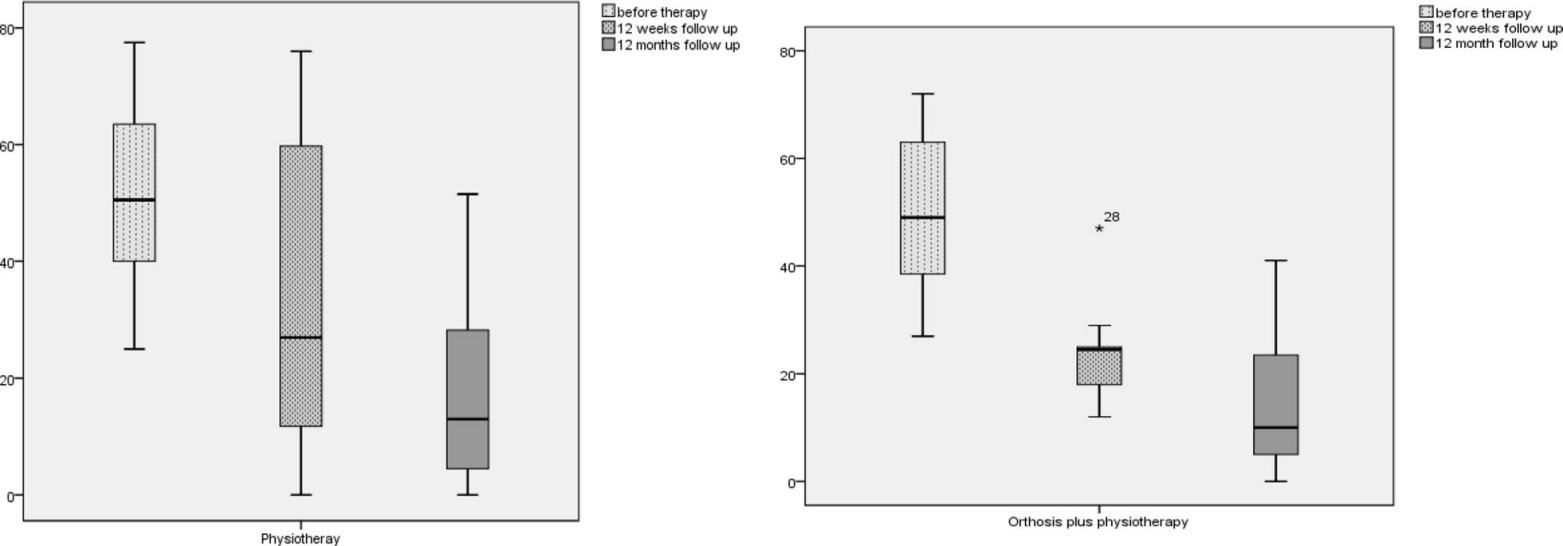
Boxplot of outcome parameter—PRTEE: baseline, 12 weeks and 12-month follow-up, left: orthosis plus eccentric strengthening exercises, right: eccentric strengthening exercises alone. {PT + O: 52.8 (SD: 16.0) to 31.3 (SD: 8.2) [*p* = .002] to 16.15 (SD: 16.1) [*p* = .000]; PT: 48.6 (SD: 19.7) to 37.6 (SD: 24.1) [*p* = .185] to 16.6 (SD: 16.1) [*p* = .000]}

The Placzek score was reduced from 8.25 (SD: 1.84) to 3.5 (SD: 2.75) [*p* = .0001] after 12 weeks for the PT + O group and from 8.1 (SD: 1.76) to 3.8 (SD: 2.98) [*p* = .000] in the PT group, as well as after 12 months in the PT + O group to 0 [*p* = .000] and in the PT group to 2.0 [*p* = .000]. The PRTEE was improved in both groups after 12 weeks (PT + O: 52.8–31.3 [*p* = .002]; PT: 48.6–37.6 [*p* = .185]) and 12 months (PT + O: 16.15 [*p* = .000]; PT: 16.6 [*p* = .000]), although the reduction at 12 weeks was not significant for the PT group (Fig. 4).

Elbow flexion graphically improved to the 12 weeks follow-up (PT + O: 133 (SD: 19) to 138 (SD: 11) [*p* = .361]; PT: 127 (SD: 19) to 130 (SD: 21) [*p* = .175]), even though it was not significant for both groups. At 12 months follow-up, a further graphic improvement in mobility was achieved for both groups (PT + O: 135 (SD: 9) [*p* = .356]; PT: 131 (SD: 17) [*p* = .734]) without reaching statistical significance. In addition, mean values of elbow extension were graphically slightly better (n.s.) in the 12-week follow-up {PT + O: 2 (SD: 4) to 1 (SD: 2) [*p* = .271]; PT: 7 (SD: 14) to 4 (SD: 14) [*p* = .175]}. In the 12 months follow-up, a further graphic improvement in mobility was achieved for the PT group (n.s.), although in the PT + O group the ROM for the extension remained constant {PT + O: 0 (SD: 0) [*p* = .689]; PT: 9 (SD: 21) [*p* = .714]}. Mean values of supination increased graphically in both groups after 12 weeks (n.s.) {PT + O: 69 (SD: 38) to 86 (SD: 5) [*p* = .073]; PT: 83 (SD: 12) to 86 (SD: 5) [*p* = .311]} and 12 month {PT + O: 86 (SD: 5) [*p* = .099]; PT: 85 (SD: 5) [*p* = .678]}.

## Discussion

The outcome of this study demonstrates that the daily use of a novel flexible wrist orthosis that unloads the wrist extensors but also daily home-based eccentric strengthening exercises alone can effectively relieve pain and improve elbow scores and grip strength. Nevertheless, the combination with a wrist orthosis seems to accelerate the short-time healing process at 3 months in regard of PRTEE and pain on the VAS.

There are several types of bandages and orthoses that are used in the treatment of LE. Struijs et al. [26] showed that an elbow brace might be useful in the initial therapy of lateral epicondylitis. However, the authors saw a short-term effect after 6 weeks, although there were no significant differences identified in all endpoints after 26 and 52 weeks. The same observation with short-time improvement in regard of PRTEE, pain and maximum painless hand strength was evaluated in the present study using a wrist orthosis. Faes et al. also examined a dynamic extensor orthosis in a RCT for 24 weeks, whereas a significant pain reduction, improved functionality of the arm and improvement in pain-free grip strength was observed with the brace treatment. This also could partially be supported with the present study. Jafarian et al. examined the clinical results of three common types of orthosis (two elbow counterforce orthosis and a wrist splint) in regard of grip strength [27]. In this study, a positive effect was seen when using the elbow strap or the elbow sleeve compared to placebo. A wrist splint had no change in pain-free or maximum grip-strength compared to the placebo orthosis, so the authors could only recommend an elbow orthosis. Nevertheless, the wrist orthosis was just a neoprene orthosis with a polyethylene bar to hold the wrist in 25° extension and had no dynamic component. Nishizuka et al. examined a forearm band versus extensor stretching exercises alone in a multicenter, randomized, controlled trial and found no significant differences between the band and non-band groups after 1, 3, 6, and 12 months [28].

Physiotherapy is one of the most important treatment options and many RCTs investigate the diversity of exercise types in combination with others. However, the results have heterogeneous evidence of effectiveness. Alfredson et al. was the first author propagating eccentric training in tendon injuries (Achilles tendinopathy) [29], although its use in LE pathology also appears to reduce the pain and improves function [30–32]. Stasinopoulos et al. [33] compared the effectiveness of eccentric training, eccentric–concentric training and eccentric–concentric training combined with isometric contraction in the treatment of lateral elbow tendinopathy. The eccentric–concentric training combined with isometric contractions achieved the largest effect in reduction of pain and improvement of function. Peterson et al. [34] achieved a greater reduction in pain but not function with a 3-month home program of concentric/eccentric forearm exercises when compared with a wait-and-see approach. Viswas et al. [35] could detect that supervised eccentric exercises improved pain and function more than friction massage in a short-term follow-up. These studies support the hypothesis that a physiotherapeutic approach with eccentric exercises has a positive impact on the resolvement of a LE. Söderberg et al. evaluated a 6-week home exercise program of eccentric exercises and a forearm band compared to a control group receiving a forearm band only. He also saw a significantly higher pain-free hand-grip and wrist-extensor strength for eccentric exercises at the end of follow-up (6 weeks) [36]. However, this deviates from our findings, as in the present study a significant reduction of pain (VAS) was observed at the 12 month follow-up, whereas after 12 weeks only a significant reduction could be achieved in the group of the wrist orthosis.

The present study has some limitations: one is the high drop-out rate of about 50% for the 12 weeks and approximately two-thirds during the 12 month follow-up. Due to the natural course of LE and the overall better and good results after 12 weeks and 12 months, the motivation for further long-time follow-up in the clinic and clinical examination maybe reduced. Overall, the drop out was similar in both study groups. One bias is the heterogeneity of the investigators and therapists, which is due to the multi-centre approach. Although patients were interviewed about their compliance during the follow-up, the potential irregular implementation of the physiotherapeutic self-exercises can influence our results.

## Conclusion

The elbow orthosis appears to accelerate the healing process with respect to the PRTEE and pain on the VAS at the 12 week follow-up, although there is an adjustment after 12 months in both groups and a significant improvement of symptoms is achieved in all endpoints.
